# Loss of ATRX, Genome Instability, and an Altered DNA Damage Response Are Hallmarks of the Alternative Lengthening of Telomeres Pathway

**DOI:** 10.1371/journal.pgen.1002772

**Published:** 2012-07-19

**Authors:** Courtney A. Lovejoy, Wendi Li, Steven Reisenweber, Supawat Thongthip, Joanne Bruno, Titia de Lange, Saurav De, John H. J. Petrini, Patricia A. Sung, Maria Jasin, Joseph Rosenbluh, Yaara Zwang, Barbara A. Weir, Charlie Hatton, Elena Ivanova, Laura Macconaill, Megan Hanna, William C. Hahn, Neal F. Lue, Roger R. Reddel, Yuchen Jiao, Kenneth Kinzler, Bert Vogelstein, Nickolas Papadopoulos, Alan K. Meeker, for the ALT Starr Cancer Consortium

**Affiliations:** 1 Laboratory for Cell Biology and Genetics, The Rockefeller University, New York, New York, United States of America; 2 Molecular Biology Program, Sloan-Kettering Institute, Memorial Sloan-Kettering Cancer Center, New York, New York, United States of America; 3 Developmental Biology Program, Sloan-Kettering Institute, Memorial Sloan-Kettering Cancer Center, New York, New York, United States of America; 4 Broad Institute of Harvard and MIT, Cambridge, Massachusetts, United States of America; 5 Department of Medical Oncology, Dana-Farber Cancer Institute, Boston, Massachusetts, United States of America; 6 Department of Microbiology and Immunology, W. R. Hearst Microbiology Research Center, Weill Medical College, Cornell University, New York, New York, United States of America; 7 Children's Medical Research Institute, Westmead, New South Wales, Australia; 8 Sydney Medical School, University of Sydney, Sydney, New South Wales, Australia; 9 Ludwig Center for Cancer Genetics and Therapeutics, Johns Hopkins Sidney Kimmel Cancer Center, Baltimore, Maryland, United States of America; 10 Howard Hughes Medical Institutions, Johns Hopkins Sidney Kimmel Cancer Center, Baltimore, Maryland, United States of America; 11 Department of Pathology, Johns Hopkins University School of Medicine, Baltimore, Maryland, United States of America; SA Pathology, Australia

## Abstract

The Alternative Lengthening of Telomeres (ALT) pathway is a telomerase-independent pathway for telomere maintenance that is active in a significant subset of human cancers and in vitro immortalized cell lines. ALT is thought to involve templated extension of telomeres through homologous recombination, but the genetic or epigenetic changes that unleash ALT are not known. Recently, mutations in the ATRX/DAXX chromatin remodeling complex and histone H3.3 were found to correlate with features of ALT in pancreatic neuroendocrine cancers, pediatric glioblastomas, and other tumors of the central nervous system, suggesting that these mutations might contribute to the activation of the ALT pathway in these cancers. We have taken a comprehensive approach to deciphering ALT by applying genomic, molecular biological, and cell biological approaches to a panel of 22 ALT cell lines, including cell lines derived in vitro. Here we show that loss of ATRX protein and mutations in the *ATRX* gene are hallmarks of ALT–immortalized cell lines. In addition, ALT is associated with extensive genome rearrangements, marked micronucleation, defects in the G2/M checkpoint, and altered double-strand break (DSB) repair. These attributes will facilitate the diagnosis and treatment of ALT positive human cancers.

## Author Summary

Telomeres, the protective elements at the ends of chromosomes, need to be maintained for cells to proliferate indefinitely. In many human cancers, the telomeric DNA is replenished by telomerase. However, a second pathway for telomere maintenance, referred to as the ALT pathway, has increasingly been recognized in human cancers. The genetic basis for activation of ALT is not known, but recent data have implicated a chromatin remodeling complex (ATRX/DAXX) and the histone variant H3.3 as players in the repression of ALT. We have examined a large panel of ALT cell lines for their genetic and cell biological features and found that loss of ATRX is a common event in the genesis of ALT lines. In addition, we document that ALT cell lines frequently undergo chromosomal changes and are impaired in their ability to detect and repair damage in their DNA. These hallmarks of ALT are expected to facilitate the detection of ALT–type tumors in the clinic and may lead to ALT–specific treatments.

## Introduction

In the absence of telomerase activity, telomeres shorten with cell division, ultimately leading to senescence. Hence, the development of human cancer is associated with an active telomere maintenance system that provides an infinite source of telomeric DNA to potentiate immortality. Although telomerase reactivation is the most common mechanism of telomeric repeat addition in cancers, a significant subset of human tumors employs a telomerase-independent telomere maintenance pathway, referred to as ALT [1].

The emerging view is that ALT maintains telomeres through homology-directed recombination (HDR) [2]. Supporting this view, ALT cells show an elevated frequency of sequence exchanges between telomeres [2]–[4], contain extrachromosomal linear and circular telomeric DNA [5]–[8], and often exhibit heterogeneously-sized telomeres [1], features consistent with hyperactive HDR. The extrachromosomal telomeric DNA has been proposed to serve as a template for the extension of telomeres by a recombination mechanism akin to Break Induced Replication (BIR) [9], [10]. In addition, ALT cells often carry altered PML bodies (ALT-associated PML bodies, or APBs) that contain telomeric DNA as well as numerous recombination factors [11]. Several proteins involved in recombination are known to be required for ALT, including the Mre11 complex, Mus81, and the SMC5/6 sumoylation pathway [6], [12]–[15].

ALT was discovered in a subset of immortalized cell lines that emerge at low frequency from human cell cultures in telomere crisis, but this pathway has increasingly been identified in human cancer. Approximately 10–15% of human cancers are suspected to use the ALT pathway based on their lack of telomerase activity in combination with certain hallmarks of ALT, such as the presence of extrachromosomal telomeric circles, APBs, and/or long and heterogeneous telomeres. Based on these criteria, ALT is relatively common in many sarcomas (osteosarcoma and some types of soft tissue sarcomas), certain endocrine tumors (pancreatic neuroendocrine tumors, paraganglioma), a subset of nervous system tumors (e.g. glioblastoma, medulloblastoma, oligodendroglioma, astrocytoma, ganglioneuroblastoma), bladder small cell carcinoma, and nonseminomatous germ cell tumors [16]–[18].

The molecular basis for the activation of the ALT pathway is not known. In vitro, the frequency of conversion to ALT is low, typically requiring many months of culturing of virally-transformed (p53/Rb deficient) human cells that have entered telomere crisis [11]. The low frequency of conversion to ALT suggests that mutations and/or infrequent epigenetic alterations are required to unleash this pathway. Indeed, in telomerase-positive mammalian cells with fully functional telomeres, telomeric recombination is stringently repressed by the TRF2, Rap1, and POT1 components of shelterin as well as by the Ku70/80 heterodimer [6], [19]–[23]. Mutations in shelterin and Ku might therefore be anticipated in ALT, a notion that is tested here.

Recently, a subset of pancreatic neuroendocrine tumors (PanNETs) were found to have ultrabright telomeric FISH signals, suggesting the presence of ALT-like long telomeres [24], [25]. These tumors also exhibited alterations in ATRX or its binding partner DAXX. Twenty-five PanNETs with ultrabright telomeric foci had *ATRX*/*DAXX* mutations or lacked nuclear ATRX/DAXX protein. In contrast, 16 PanNETs with apparently normal ATRX/DAXX lacked the aberrant telomeric staining patterns. The same correlation between inactivation of the ATRX pathway and the ALT-like phenotype was observed in pediatric glioblastoma and several other cancers [16], [26].

ATRX and DAXX act together in a replication-independent chromatin assembly pathway that deposits the histone variant H3.3 at telomeres and perhaps at other G-rich repetitive elements [27]–[31]. The function of ATRX/DAXX and histone H3.3 at telomeres is not yet clear, but diminished ATRX function has been documented to increase TTAGGG repeat containing telomeric transcripts (referred to as TERRA [32]), reduce telomeric loading of HP1α, and cause modest levels of telomere dysfunction in mouse ES (but not NIH3T3) cells as gleaned from the localization of γ-H2AX at chromosome ends [29], . ATRX deficiency also leads to a defect in sister chromatid cohesion and aberrant mitoses, culminating in the formation of micronuclei, lobulated nuclei, and chromatin bridges [33]–[35].

Because functional assessment of ALT is not possible in tumor specimens, further tests for the association of ALT with ATRX/DAXX deficiency requires analysis of in vitro immortalized cell lines. Here we describe the results of a comprehensive effort to characterize the genetic alterations and associated phenotypes of 22 human ALT cell lines. We found loss of ATRX in 90% of in vitro immortalized ALT lines, suggesting that inactivation of ATRX is a major step in generating the ALT phenotype. We also document that ALT cell lines are characterized by extensive genomic instability, formation of micronuclei, an aberrant G2/M checkpoint, and abnormal DSB repair kinetics.

## Results

### Most human ALT cell lines lack ATRX

We assembled a panel of 22 human ALT cell lines (Table 1), nineteen of which originated from in vitro immortalization experiments and four of which were derived from human tumors (three osteosarcomas and one lung adenocarcinoma). Many of the in vitro immortalized lines expressed SV40- or HPV-derived oncoproteins. Seven cell lines originated from a single individual with cystic fibrosis (JFCF-6 series); an additional five cell lines were established from a Li-Fraumeni patient (IIICF series). We confirmed the absence of telomerase activity by TRAP assay (data not shown) and the presence of extra-chromosomal telomeric C-circles, supporting the interpretation that these were ALT cells [8], [17] (Table 1; Figure S1).

**Table 1:** Description of ALT lines used in this study and summary of results.

Cell Line	Tissue Type	Disease (a)	Transformation	C-circles (b)	ATRX protein (c)	ATRX gene (d)	DAXX protein (c)	DAXX gene	H3F3A gene	Micro-nuclea-tion (e)	G2/M checkpoint initiation (f)	G2/M checkpoint maint (g)	DSB repair (h)	DSB repair (i)	% cells with >10 53BP1 foci (j)	Reference
JFCF-6/T.1D	Jejunal fibroblast	CF	SV40 ER	100*	**undetectable**	**deletion**	normal	wild type	wild type	8±1.4	**27±4**	**69±6**	0.1±5	normal	44	60
JFCF-6/T.1L	Jejunal fibroblast	CF	SV40 ER	303±54	**undetectable**	**deletion**	normal	wild type	wild type	**12±0.9**	**46±5**	**81±4**	*nt*	*nt*	**72**	(k)
JFCF-6/T.1M	Jejunal fibroblast	CF	SV40 ER	76±7	**undetectable**	wild type	normal	wild type	wild type	**16±0.5**	*73±3*	**39±12**	*nt*	*nt*	**62**	(k)
JFCF-6/T.1Q	Jejunal fibroblast	CF	SV40 ER	120±8	**undetectable**	wild type	normal	wild type	wild type	**10±3.9**	**8±18**	**56±19**	**27±2**	**deficient**	51	(k)
JFCF-6/T.1R	Jejunal fibroblast	CF	SV40 ER	42±7	**undetectable**	**deletion**	normal	wild type	wild type	9±3.8	**60±5**	*nt*	**19±15**	**deficient**	53	11
JFCF-6/T.1J/5H	Jejunal fibroblast	CF	SV40 ER	217±57	**undetectable**	**deletion**	normal	wild type	wild type	9±3.9	**4±1**	**64±7**	**14±15**	**deficient**	31	60
JFCF-6/T.1J/1-3C	Jejunal fibroblast	CF	SV40 ER	82±16	**undetectable**	**deletion**	normal	wild type	wild type	**10±1.1**	**1±4**	*nt*	**40±8**	**deficient**	55	38
IIICF/a2	Breast fibroblast	LF	spontaneous	402±44	**undetectable**	wild type	normal	wild type	wild type	**17±5.6**	*60±10*	*nt*	*nt*	*nt*	**88, 87**	11
IIICF/c	Breast fibroblast	LF	spontaneous	125±20	**undetectable**	wild type	normal	wild type	wild type	**14±3.0**	89±1	**58±8**	*nt*	*nt*	**73, 71**	61
IIICF-T/A6	Breast fibroblast	LF	SV40 ER	199±5	**undetectable**	wild type	**undetectable**	wild type	wild type	**11±4.1**	**28±6**	*nt*	*nt*	*nt*	**66, 68**	61
IIICF-T/B3	Breast fibroblast	LF	SV40 ER	278±45	**undetectable**	**deletion**	normal	wild type	wild type	**16±1.3**	82±2	**79±3**	*nt*	*nt*	**74, 78**	61
IIICF-E6E7/C4	Breast fibroblast	LF	HPV	101±24	**abnormal IF**	wild type	normal	wild type	wild type	**24±1.2**	*83±8*	*nt*	*nt*	*nt*	**81, 85**	61
IVG-BF.LXSN	Breast fibroblast	LF	spontaneous	395±89	**abnormal IF**	wild type	**low level/abnormal IF**	wild type	wild type	**27±3.7**	83±2	*nt*	*nt*	*nt*	**71, 77**	(l)
LFS-05F-24	Skin fibroblast	LF	spontaneous	142±8	normal	wild type	normal	wild type	wild type	**20±5.9**	*77±5*	*nt*	*nt*	*nt*	**68, 73**	8
WI38-VA12/2RA	Fetal lung fibroblast	n/a	SV40	67±11	**undetectable**	wild type	normal	wild type	wild type	7±2.9	*48±10*	**67±5**	**50±11**	**deficient**	21, 37	61
SUSM-1	Embryonic liver fibroblast	n/a	chemical	78±22	**undetectable**	wild type	normal	wild type	wild type	**24±3.6**	*92±1*	*nt*	**41±5**	**deficient**	46, 56	61, 62
GM847	Skin fibroblast	LN	SV40	117±5	**undetectable**	wild type	normal	wild type	wild type	6±1.0	*44±9*	*nt*	**54±5**	**deficient**	17, 38	61
MeT-4A	Pleural mesothelium	n/a	SV40 ER	50±7	normal	wild type	normal	wild type	wild type	**15±6.5**	*nt*	**58±3**	**31±8**	**deficient**	17, 23	61
SaOS-2	Bone	OS	n/a	661±90	**undetectable**	wild type	**low level**	wild type	wild type	**16±4.3**	71±5	*nt*	*nt*	*nt*	**72**	1
G292	Bone	OS	n/a	112±12	**abnormal IF**	wild type	**abnormal IF**	wild type	wild type	**12±1.6**	84±2	**80±7**	**44±14**	**deficient**	22. 22	1
U-2 OS	Bone	OS	n/a	104±38	**undetectable**	**deletion**	normal	wild type	wild type	7±2.5	95±1	99±1	**33±1**	**deficient**	7, 9	1
SK-LU-1	Bronchial epithelium	Lung adenocarcinoma	n/a	323±31	normal	wild type	normal	wild type	wild type	**16±3.1**	*6±18*	*nt*	*nt*	*nt*	**75**	1

**Figure 1:**
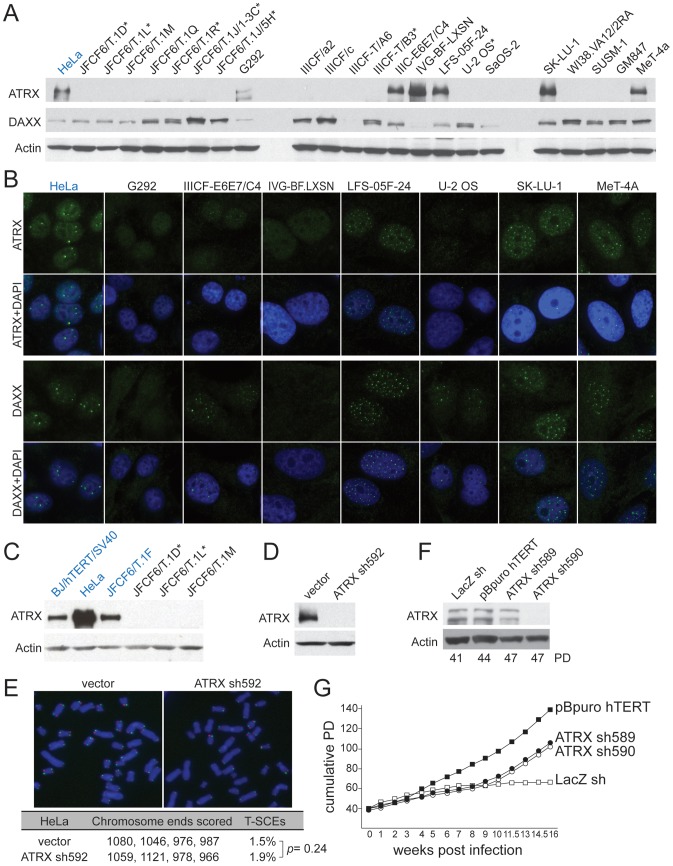
Deficiency in ATRX/DAXX correlates with ALT. A, Immunoblot for ATRX and DAXX in the indicated ALT lines. ATRX was detected with A301-045 (Bethyl Labs). DAXX was detected with A301-353A (Bethyl Labs). Asterisk: cell lines with deletions in the *ATRX* gene (see Table 1 and Table S2). HeLa cells (blue) were used as a positive control. B, IF for ATRX and DAXX in the indicated cell lines. HeLa cells (blue) are used as a positive control. C, Immunoblot for ATRX in additional non-ALT, positive control cells (blue) relative to the indicated ALT lines. D, Immunoblot showing >90% reduction of ATRX protein in HeLa cells expressing ATRX shRNA592. E, T-SCEs, a measure for telomeric recombination, assessed by Chromosome Orientation (CO)-FISH on metaphases harvested from HeLa cells expressing vector or ATRX shRNA592. The average percentage of T-SCEs from four independent experiments is shown, with a *p* value derived from a two-tailed, unpaired *t* test. F, Immunoblot showing >90% reduction of ATRX protein in cells expressing ATRX shRNA590 but not with shRNA589. G, Growth curves showing immortalization of SV40-transformed BJ fibroblasts infected with an hTERT expressing retrovirus but no immortalization after infection with the effective (sh590) and ineffective (sh589) shRNAs to ATRX.

(a) CF, cystic fibrosis; HPV, human papillovirus 16 E6 and E7; LF, Li-Fraumeni syndrome; LN, Lesch-Nyhan syndrome; OS, osteosarcoma; SV40 ER, simian virus 40 early region.

(b) Values are averages of triplicate assays (±SD) with JFCF-6/T.1D set to 100 in each assay and the other values expressed relative to this standard. BJ and HeLa values are below 2.

(c) Bold: aberrant ATRX or DAXX protein in western and/or IF. ATRX western results are from three independent immunoblots. Examples of abnormal IF are given in Figure 1b.

(d) Bold: deletions in ATRX. See Table S1 for details.

(e) Values represent mean % cells with micronuclei and SDs from three independent experiments. See Figure 4. Values for HeLa and BJ/SV40 are <8%. Bold: >10% of cells with micronuclei.

(f) Bold: abnormal G2/M checkpoint initiation (<70% reduction in mitotic index 1 hr after 10 Gy IR). See Figure 5A. Italic: uninterpretable due to low mitotic index. hTERT-RPE (positive control) 89% and ATM-/- GM5849 46%.

(g) Bold: abnormal G2/M checkpoint maintenance (<90% reduction in mitotic index 16 hr after 4.5 Gy IR). See Figure 5b. Italic: low mitotic index at 16 hr in noc. Values for HeLa and BJ/SV40 were 98±2% and 96±3%, respectively.

(h) Bold: slow DSB repair kinetics. See Figure 6A and Figure S8. After 0.5 Gy IR, cells with >10 53BP1 foci were scored at 1 and 24 hr and without IR. Values represent (% at 24 hr)-(% no IR)/(% at 1 hr)-(% no IR). nt: not tested.

(i) Bold: abnormal residual DNA fragmentation 24 hr post IR. See Figure 6B. nt: not tested.

(j) Bold: greater than 60% of cells containing >10 spontaneous 53BP1 foci.

(k) A. Englezou, P. Bonnefin, R. Reddel, unpublished data.

(l) J. Plowman, L. Huschtscha, R. Reddel, unpublished data.

The ALT cell lines and non-ALT controls (hTERT-immortalized SV40-transformed BJ fibroblasts and the telomerase-positive HPV-E6/E7 expressing HeLa cervical tumor cell line) were tested for the expression of ATRX and DAXX (Figure 1A–1C and data not shown). Repeated immunoblots on independently harvested whole cell extracts indicated that among the 22 ALT cell lines, only six (G292, IIICF-E6E7/C4, IVG-BF.LXN, LFS-05F-24, SK-LU-1, and MeT-4A), contained detectable full length ATRX protein and one of these (IVG-BF.LXN) appeared to lack DAXX. Of the six cell lines expressing ATRX, three (G292, IIICF-E6E7/C4, and IVG-BF.LXN) showed very low immunofluorescence signals for ATRX and/or DAXX (Figure 1B), whereas the other three lines displayed the punctate nuclear staining pattern for ATRX and DAXX expected for this PML body component [36], [37]. Thus, 19 out of 22 ALT lines had an alteration in the expression of ATRX and/or DAXX. As each of the seven JFCF-6-derived ALT lines lacked detectable ATRX, we considered the possibility that the parental JFCF-6 culture might have been deficient in ATRX expression. However, a non-ALT JFCF-6 line contained ATRX (Figure 1C), indicating that the lack of ATRX in the other JFCF-6 lines was potentially correlated with the ALT phenotype.

All ALT cell lines were tested for genetic changes in *ATRX*, *DAXX*, and *H3.3* (*H3F3A*) using primers designed to PCR-amplify all exons from these genes for Sanger sequencing (Table 1; Table S1). Failure to amplify multiple consecutive exons was interpreted as a deletion event only when flanking exons did amplify. By that criterion, 6 cell lines (JFCF-6/T.1D, JFCF-6/T.1L, JFCF-6/T.1R, JFCF-6/T.1J/5H, JFCF-6/T.1J/1-3C, and IIICF-T/B3) harbored large deletions in the *ATRX* gene that ranged from 4 to 26 exons in size (Table 1; Table S1).

Sequencing of genes that did not harbor detectable deletions revealed no potentially inactivating genetic changes. Nine of those cell lines lacked detectable ATRX protein suggesting that genetic alterations not detectable by Sanger sequencing, such as translocations, promoter or splicing mutations, or epigenetic changes may underlie their lack of expression. No genetic alterations were detected in *DAXX* or the *H3F3A* gene encoding H3.3 (Table 1).

These data provide strong evidence that loss of ATRX expression is involved in either the initiation or maintenance of the ALT pathway in human cells. To test whether deficiency in ATRX was sufficient to induce the telomere-telomere recombination typical of ALT cells, we used an shRNA to deplete ATRX from HeLa cells and used Chromosome-Orientation FISH (CO-FISH) to measure sequence exchanges between sister telomeres (Telomere Sister Chromatid Exchanges or T-SCEs). Despite repression of ATRX by more than 95%, the frequency of T-SCEs was not significantly altered (Figure 1D and 1E). In addition, repression of ATRX or DAXX in SV40-transformed BJ fibroblasts failed to induce escape from crisis (Figure 1F and 1G; Figure S2), whereas expression of hTERT readily immortalized the cells (Figure 1G; Figure S2). After a prolonged period in crisis (6 weeks), immortal clones emerged from the culture in which ATRX expression was suppressed with ATRX sh590. However, a parallel culture in which ATRX was not suppressed efficiently (ATRX sh589) also yielded an immortal subpopulation (Figure 1F and 1G) and in both cases, the immortal cells expressed telomerase and lacked telomeric C-circles (Figure S2A and S2B), indicating that they had not activated ALT. These observations argue that this level of ATRX/DAXX suppression is not sufficient to activate the ALT pathway.

### No overt changes in shelterin or 299 potentially ALT relevant genes

The lack of induction of ALT by shRNA-mediated suppression of ATRX and DAXX prompted us to inspect the ALT cell line panel for changes in the status of genes, pathways, and proteins with possible relevance to the activation of ALT. Because HDR is normally repressed at telomeres by shelterin, we examined the expression of all six shelterin components in the ALT lines by repeated immunoblotting (Figure S3). Using this approach on all 22 ALT lines failed to reveal a consistent change in the abundance or appearance of the six shelterin proteins. In addition, exome sequencing (see below) failed to reveal mutations in the genes encoding TRF1, TRF2, Rap1, POT1, TIN2, and TPP1.

A second entity associated with telomeres is the telomeric RNA, TERRA [32]. As mouse ATRX deficient cells were reported to have elevated TERRA levels and selected ALT cells were previously found to have higher levels of TERRA [31], [38], we examined the expression of this RNA in the ALT lines. Quantitative Northern analysis confirmed that the ALT lines generally have higher TERRA levels than telomerase-positive cells, but some ALT lines (e.g. JFCF-6/T.1D and IIICF-E6E7/C4) approximated the levels of TERRA found in BJ/hTERT/SV40 and HeLa cells (Figure S4). There was no clear correlation between increased TERRA and ATRX status since the cell lines with apparently normal ATRX/DAXX (LFS-05F-24, SK-LU-1, and MeT-4A) had TERRA levels comparable to, or higher than, cell lines lacking ATRX (e.g. JFCF-6/T.1D, JFCF-6/T.1R, and JFCF-6/T.1L) (Figure S4).

In addition to shelterin and TERRA, we analyzed 299 genes with potential relevance to telomeres and the ALT pathway. This gene set included genes encoding proteins involved in telomere maintenance, DNA damage repair and signaling, chromosome duplication and segregation, and cell cycle regulation (Table S2). The coding regions of these genes were analyzed by massively parallel 454 sequencing on genomic DNA derived from 14 of the 22 ALT lines. We focused on ALT lines generated *in vitro* as opposed to tumor-derived ALT lines to exclude genetic changes selected for during tumorigenesis. The JFCF-6 and IIICF ALT lines are derived from two individuals, allowing identification of SNPs specific to the individual as well as potential ALT-correlated changes. After excluding known SNPs, the remaining sequence data were filtered to exclude apparent SNPs present in less than 25% of the reads. The rare SNPs that eluded the threshold filter affected 20 genes (Table S3). Within the JFCF-6 and IIICF cell line sets, a nucleotide change in multiple members of either set was interpreted as being present prior to the acquisition of the ALT phenotype, and thus not relevant. The variable occurrence of these SNPs in the multiple ALT lines derived from one individual (e.g. only two of the five IIICF derived lines have the BLM K323R) is likely due to chromosome losses occurring during or after telomere crisis. The florid chromosome instability exhibited by these cell lines (see below) makes this an appealing and parsimonious explanation. Some of these SNPs were also found in other cell lines (e.g. MDC1 C1559G in LSF-05F-24 and IVG-BF.LXSN) and deemed irrelevant based on their presence in the IIICF and/or JFCF-6 lines prior to the induction of ALT. Based on these criteria, no ALT specific change was observed in the five IIICF-derived ALT lines or in IVG-BF.LXN. Three of the JFCF-6 derived ALT lines had a specific change that could not be attributed to a pre-existing SNP (A436S in Suv420H2, S553F/S470F in PRMT5, and D2579V in Ki67; Table S3). Since these genes were not affected in the other 13 ALT lines analyzed, they are unlikely to be critical to ALT. Similarly, LSF-05F-24 contained changes in five genes that are not affected in the other 13 cell lines. Since none of these alterations were found in a substantive fraction of the ALT cell lines, it is unlikely that these genes represent clear contributors to the ALT phenotype.

### Extensive genome rearrangements in ALT

**Figure 2:**
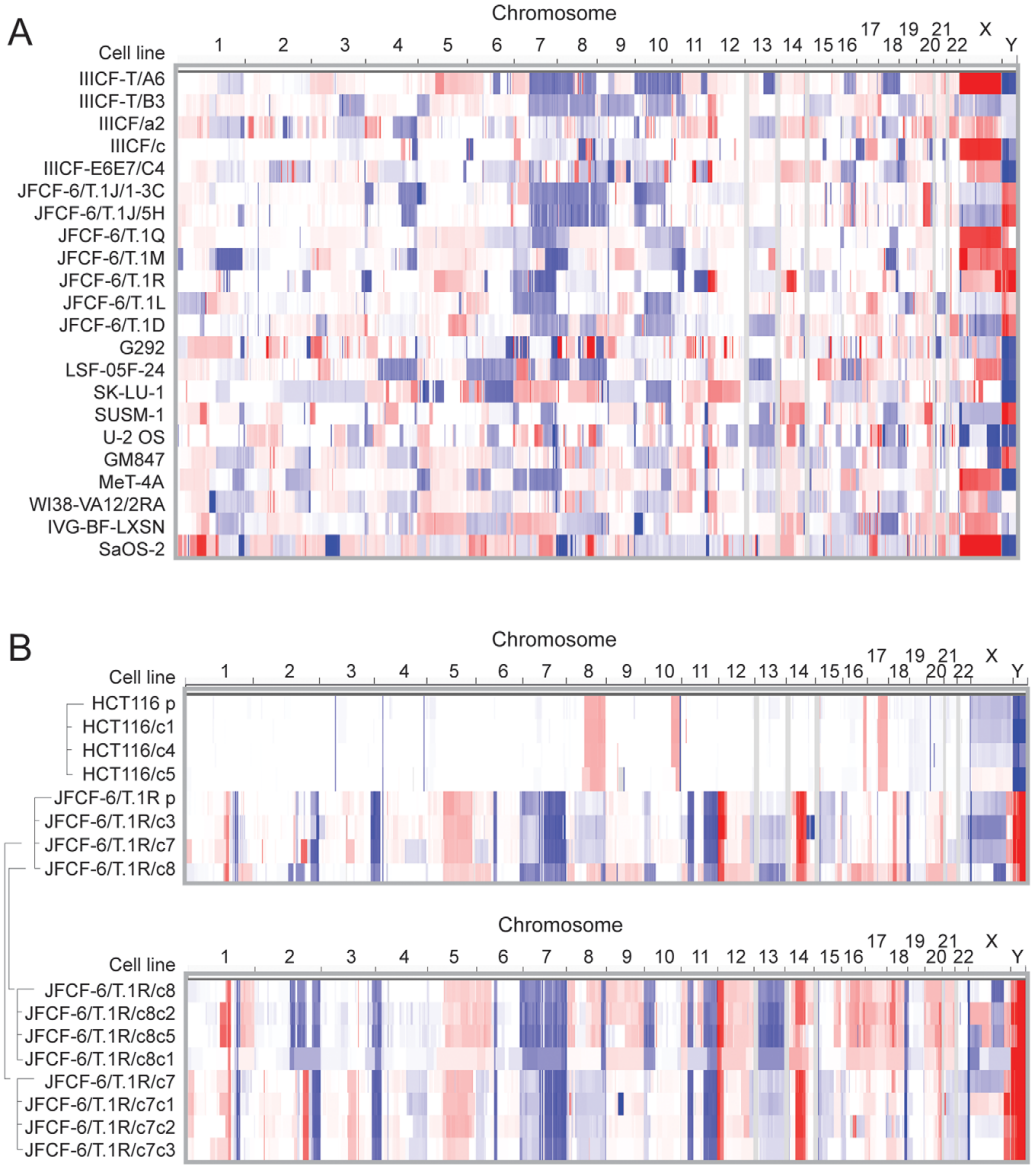
Copy number analysis showing extensive genome rearrangements in ALT lines. SNP array copy number results are shown for (A) the ALT cell lines and (B) the 1st and 2nd generation subclones derived from JFCF-6/T.1R (indicated by the brackets) and subclones derived from the telomerase-positive HCT116 control. Segmented copy number data is shown for each chromosome, by genomic position in columns and by cell line in rows. The color scale ranges from red (amplification; log2 copy number ratio of 1.5) through white (neutral; 2 copies in diploid lines, log2 ratio of 0) to blue (deletion; log2 ratio of −1.5).

As ALT-relevant genetic alterations might be identified from recurrent regions of gene copy number changes, we analyzed the ALT cell line panel using Affymetrix 6.0 single nucleotide polymorphism (SNP) arrays to identify regions of recurrent copy number alteration. When we examined either individual cells (Figure 2A and 2B) or the entire set as a group (GISTIC analysis, Figure S5), we found that the number and complexity of these gains and losses were at least as extensive as the alterations in human cancers with genome instability [39]. When we considered all of the ALT cell lines as a group, we identified 18 regions of significant focal copy number gain, and many more regions of copy number loss (Figure S5).

**Figure 3:**
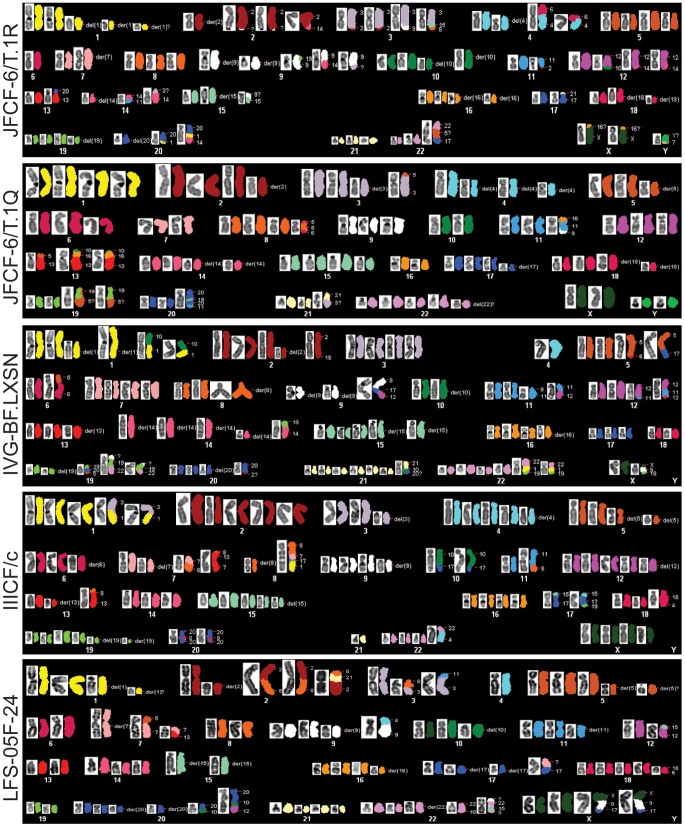
Abnormal karyotypes in ALT lines. Representative metaphases from 5 cell lines showing subtetraploid karyotype with high number of rearranged chromosomes (up to 60%). Structural rearrangements are labeled as following: del(Z)- deletion of chromosome Z; der(Z) - multiple aberrations within single chromosome Z; chromosome, denoted with two or more numbers indicate rearrangement involving two or more partners.

The genome rearrangements in the ALT lines are a likely consequence of the telomere dysfunction these cells experienced prior to their immortalization. Consistent with this idea, SKY analysis of five ALT lines (JFCF-6/T.1R, JFCF-6/T.1Q, IVG-BF.LXN, IIICF/c, and LFS-05F-24) showed frequent non-reciprocal translocations, deletions, complex rearrangements, and hyper-triploid chromosome numbers (Figure 3), all of which can be caused by telomere dysfunction [40], [41]. Previous cytogenetic analysis of two ALT cell lines (Saos-2 and ZK-58) also indicated frequent translocations, deletions, and complex rearrangements [42].

In cells that have been immortalized by telomerase activation, the telomere-driven genome instability is largely dampened, resulting in a rearranged but now relatively stable karyotype [43], [44]. We asked whether ALT similarly stabilizes the genome by examining two generations of clonal descendants of the JFCF-6/T.1R ALT line. Although the clones exhibited features in common with the parental cell line, each of the clones showed new regions of copy number gain or loss (Figure 2B). For instance, a new gain of a segment on chromosome 2q was observed in one single cell clone of the parental line (and all the clones derived from it), and in another clone (and its derivatives) an overlapping segment showed a new deletion. Many other changes, including examples on chromosomes 6, 12, 14, 15, 16 and 21, showed obvious new gains or losses as compared to the parental clone, with independent clones showing different alterations. In contrast, we found only rare clonal alterations in single cell clones derived from the telomerase-expressing HCT116 cell line. These observations suggest that the JFCF-6/T.1R and perhaps other ALT lines have ongoing genome instability as well as highly rearranged genomes.

**Figure 4:**
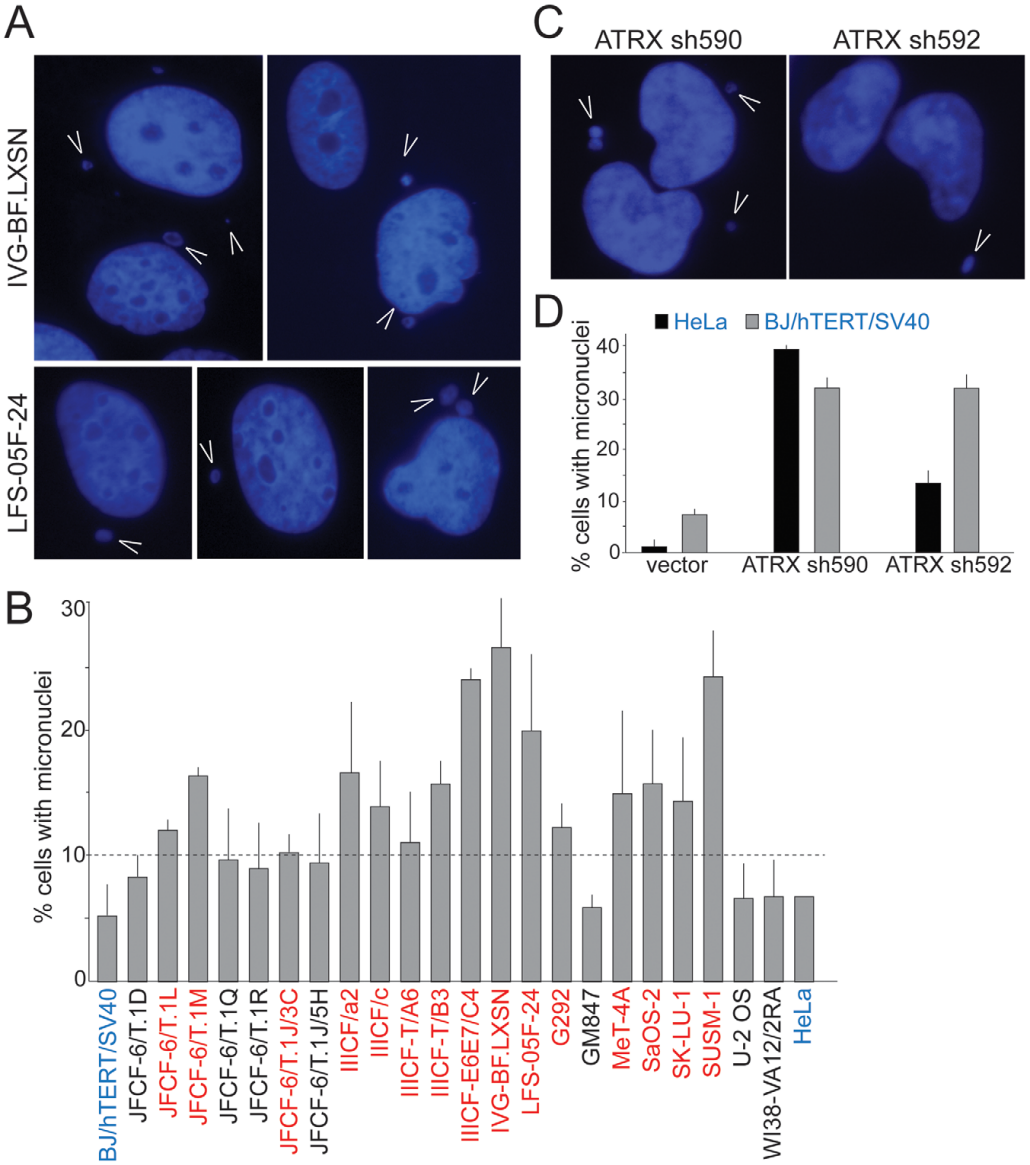
Frequent micronucleation in ALT cells may be attributable to loss of ATRX. A, Examples of micronuclei (arrowheads) in two ALT cell lines. Blue: DAPI stain for DNA. B, Graph showing micronucleation frequencies in the ALT cell lines and two telomerase positive controls (blue). Values are means ±SD from three experiments (>100 nuclei each). Red: cell lines with >10% micronucleation frequency (dashed line). HeLa cells were analyzed once. C, Examples of micronuclei (arrowheads) in HeLa cells expressing two independent ATRX shRNAs (see Figure 1 for immunoblots). D, Graph showing micronucleation frequencies in two telomerase positive cell lines infected with vector or the indicated ATRX shRNAs. Values are means ±SEM from two experiments (200 nuclei each).

We considered that ongoing genome instability in the ALT lines could be due to defects in mitosis and/or dicentric chromosomes resulting from telomere dysfunction and ATRX deficiency. Defects in mitosis and dicentric chromosomes can give rise to lagging chromosomes that can form micronuclei, which were recently shown to engender massive genomic alterations [45]. We therefore examined the ALT lines for the spontaneous occurrence of micronuclei. Fifteen of the 22 ALT lines showed a high frequency (10–30%) of cells with micronuclei (Table 1; Figure 4A and 4B). As expected, SV40-transformed hTERT-expressing BJ fibroblasts and HeLa cells showed the low level of spontaneous micronucleation (<8%) previously noted in other transformed human cells [46]. Micronucleation frequencies of up to 15% were previously only observed in cells experiencing high levels of DNA damage ensuing from γ-irradiation, in genetic contexts associated with high levels of spontaneous DNA damage, and in genomically unstable tumor cell lines (e.g. [47], [48]; Neill Ganem and David Pellman, pers. comm.). Thus, many ALT cell lines show an unusually high frequency of micronucleation, indicative of ongoing genome instability. The micronucleation phenotype of ALT lines is likely to be in part due to the absence of ATRX since depletion of ATRX with two shRNAs induced the formation of micronuclei in BJ/hTERT/SV40 and HeLa cells (Figure 4C and 4D), consistent with a previous report on the induction of lobulated nuclei after ATRX depletion [35].

### G2/M checkpoint function and DSB repair kinetics in ALT lines

**Figure 5:**
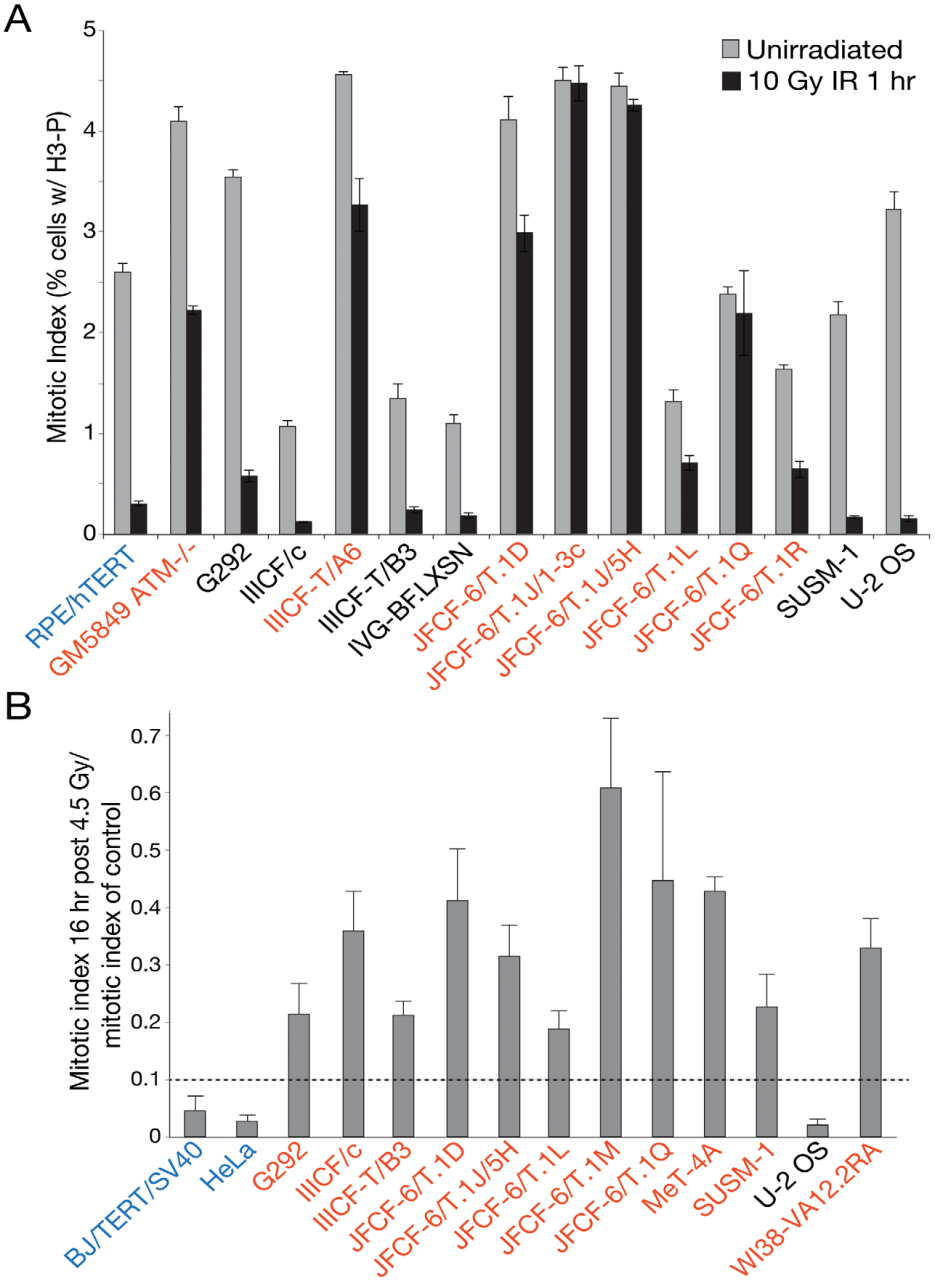
Many ALT lines have a defect in the G2/M checkpoint initiation and/or maintenance. A, Bar graph depicting the results of an assay for the initiation of the G2/M checkpoint at 1 hr after irradiation of the indicated cell lines. RPE/hTERT is a positive control (in blue). Mean ± SD for triplicate experiments are shown. B, Bar graph depicting the results of an assay for the maintenance of the G2/M checkpoint at 16 hr after IR. BJ/hTERT/SV40 is a positive control (in blue). Mean ± SD for triplicate experiments are shown. See Table 1 for summary. Cell lines in red have a defect in the G2/M checkpoint.

The signs of ongoing genome instability in ALT cells led us to examine the functionality of DNA damage checkpoints and DSB repair. As the ALT lines were expected to have an impaired G1/S DNA damage checkpoint due to their p53 deficiency, we focused our efforts on the G2/M checkpoint. Entry into mitosis is blocked upon activation of the ATM and/or ATR signaling pathways. ATM signaling is primarily responsible for the initiation of the checkpoint, whereas ATR signaling ensures the maintenance of the arrest [49]. To determine the efficiency of initiation and maintenance of the G2/M checkpoint, subconfluent cells were subjected to ionizing radiation, and the mitotic index was determined by flow cytometric quantitation of histone H3 (S10) phosphorylation. One hour after treatment with 10 Gy ionizing radiation (IR), the mitotic index of control hTERT-immortalized Retinal Pigment Epithelial cells (RPE/hTERT) was reduced approximately ten-fold (from 2.7% to 0.3%), whereas reduction in the mitotic index of ATM-deficient GM5849 cells was less than two-fold (from 4.1% to 2.3%) (Table 1; Figure 5A). Of fourteen ALT lines that had a sufficiently high mitotic index to yield interpretable results, half showed a severe defect in G2/M checkpoint initiation (Figure 5A; Table 1). In six ALT lines, the checkpoint defect was more severe than that of ATM-deficient cells. Qualitatively similar results were observed in response to low dose (1 Gy) IR treatment, with the same cell lines exhibiting defects as at the high dose (data not shown). Similarly, 10 of 11 ALT lines tested displayed a deficiency in the maintenance of the G2/M checkpoint, monitored at 16 hr after γ-irradiation with 4.5 Gy (Table 1; Figure 5B). Some of the ALT lines were proficient in the initiation of the checkpoint but failed to maintain it (e.g., IIICF/c). Collectively, we observed impaired G2/M checkpoint function in 11 out of 17 ALT lines tested. This defect in the G2/M checkpoint is not seen in established non-ALT human cell lines. For example, normal G2/M arrest following γ-irradiation is observed in HT1080, HCT116, IMR90 (JHJP, unpubl. data), 293T [50], MCF7, and genetically complemented HCC1937 cells [51]. The decrement in the G2/M checkpoint did not reflect a global impairment of DNA damage response (DDR) signaling in the ALT cells, since phosphorylation of CHK2, an outcome of ATM signaling, was readily apparent upon IR treatment in all cell lines, but undetectable in GM5849 (ATM-deficient) cells (Figure S6). Indeed, CHK2 phosphorylation was evident in several of the ALT cell lines prior to irradiation, consistent with the finding that the ALT phenotype is associated with chronic genotoxic stress (Figure S6).

**Figure 6:**
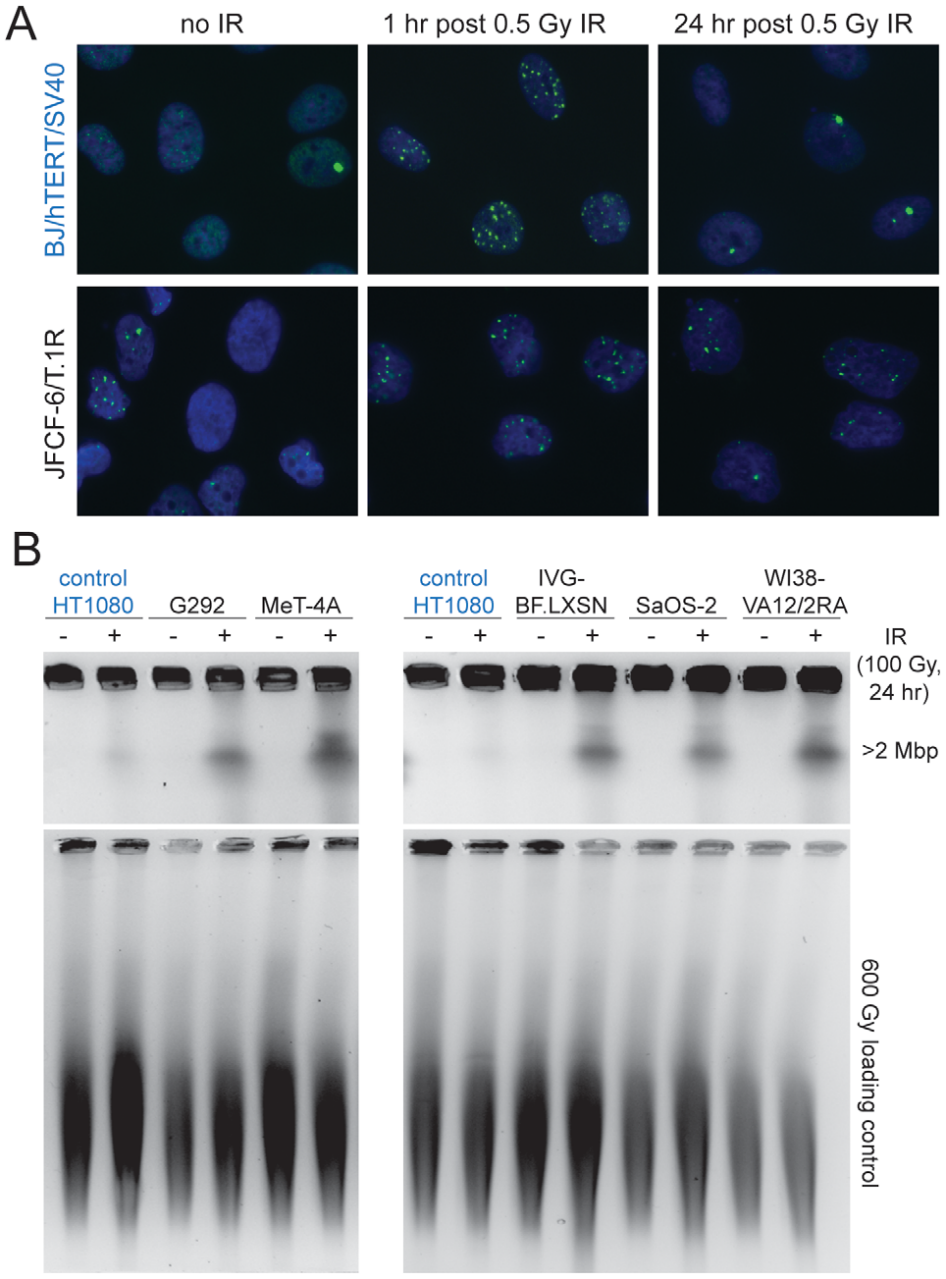
Deficiency in DSB repair in ALT lines. A, Example of assay for 53BP1 foci in the positive control (BJ/hTERT/SV40, blue) (top) and an ALT line (bottom). Cells were treated as indicated above the panels and stained for DNA with DAPI (blue) in conjunction with IF for 53BP1 (green). Note the higher level of 53BP1 foci in non-irradiated ALT cells and incomplete DSB repair at 24 hr after IR. B, Example of PFGE-assay for DSB repair in the indicated cell lines. Cells were treated with 100 Gy IR 24 hr before harvesting and residual DSBs were evaluated based on the DNA fragments released from agarose embedded cells into a PFGE gel. Plugs were treated with 600 Gy to fragment the DNA and run in parallel to serve as a control for loading. DNA was stained with ethidium bromide after electrophoresis. HT1080 (fibrosarcoma cell line) was used as a positive control (in blue).

In addition to an abnormal G2/M checkpoint, many of the ALT cell lines showed unusually slow DSB repair kinetics. To assay this aspect of the DDR, cells were γ-irradiated with 0.5 Gy and the fraction of cells with 10 or more 53BP1 foci was scored after 1 and 24 hr as well as in non-irradiated controls (Figure 6A; Figure S7; Table 1). As expected, non-ALT cell lines (HeLa and hTERT immortalized SV40-transformed BJ) showed a nearly complete disappearance of the IR-induced DNA damage foci at 24 hr. In contrast, 11 out of 12 ALT cell lines retained a significant fraction of the induced 53BP1 DNA damage foci after a day (Table 1; Figure 6A). This result was corroborated with a pulsed field gel electrophoresis (PFGE)-based gel assay for the disappearance of IR-induced DNA fragmentation after 24 hr (Figure 6B; Table 1). We also noted that approximately half of the ALT cell lines had a high basal level of cells containing 10 or more 53BP1 foci, further confirming that ALT cells experience ongoing genome damage (Table 1). These spontaneous 53BP1 foci could be the result of both genome-wide DNA breaks and the presence of dysfunctional telomeres [52]. Their deficiency in the G2/M checkpoint, combined with the absence of a functional p53 pathway likely explains why these cells proliferate despite severe genome damage.

## Discussion

Here we report that ATRX is either undetectable or severely depleted from PML bodies in ∼90% of human ALT cell lines tested, including ALT lines derived in vitro. This result establishes a strong correlation between the initiation or maintenance of ALT and deficiency in the ATRX/DAXX pathway. In a subset of cases, the loss of ATRX is due to large deletions within the ATRX gene, further underscoring the relevance of ATRX status to ALT. We expect that alterations in ATRX/DAXX in cells and tumors will be a useful indicator of the presence of the ALT pathway. However, manipulating ATRX/DAXX expression failed to unleash ALT, suggesting that deficiency of these proteins is not sufficient for the ALT phenotype and pointing to the need to identify the cooperating (epi-) genetic changes. In addition, a key issue for further investigation is the mechanism by which the loss of ATRX/DAXX and concomitant lack of deposition of H3.3 into telomeric chromatin might allow or promote telomere recombination. We note that it remains possible that the ATRX/DAXX pathway has an indirect effect, for instance by repressing genes that promote telomere recombination.

Our data have uncovered a hitherto unappreciated level of genome instability in ALT cells. All ALT lines, including cell lines that arose from in vitro immortalization experiments, harbor scrambled genomes and have signs of ongoing genome instability. The ALT lines have frequent micronuclei, a high basal level of DNA damage foci, and elevated checkpoint signaling in absence of exogenous damage. Furthermore, in one ALT cell line, repeated subcloning established frequent copy number alterations. These features of ALT raise the possibility that tumors employing this pathway will have unique vulnerabilities that could offer clinical utility, such as has been shown for cells lacking BRCA1 that are sensitive to PARP inhibition [53]. This possibility underscores the importance of establishing a signature for the ALT phenotype.

In this context, it is notable that G2/M checkpoint deficiency emerged as a prominent attribute of many ALT cells in this study. It is likely that the diminished G2/M checkpoint response allows these cells to proliferate, despite a considerable burden of DNA damage both at telomeres and elsewhere in the genome. G2/M checkpoint deficiency is an example of a potential vulnerability unique to ALT cells. Indeed, G2/M checkpoint inhibitors to enhance efficacy of clastogenic therapies are currently being developed and evaluated in clinical trials [54].

The altered G2/M response could not be ascribed to mutations in known components of the ATM or ATR signaling pathways. Furthermore, DNA damage signaling was demonstrably intact since CHK2 phosphorylation could be induced by exogenous DNA damage. The implication of ATRX and DAXX in the ALT phenotype may have some relevance to the checkpoint defects observed, as the involvement of chromatin remodelers in the DDR becomes increasingly clear [55]. Nevertheless, identification of the mechanisms underlying G2/M checkpoint dysfunction in ALT cells is warranted given the data presented here.

Our results would appear to exclude the possibility that the ALT cells arise from a single dominant mutation in addition to p53/Rb loss. Loss of ATRX/DAXX function appears to be required for ALT but it seems likely that a defect in the G2/M checkpoint is also needed in order for the cells to proliferate, given their high level of spontaneous DNA damage. The view that multiple steps are required to allow ALT-mediated immortalization is consistent with the low frequency by which it arises, even in SV40- or HPV-transformed cells.

## Materials and Methods

### Cell lines

ATL cell lines are described in Table 1. CellBank Australia provided cell line quality control. All cell lines were verified by 16-locus STR profiling and confirmed to be free of Mycoplasma species, or were analyzed within 10 population doublings of being obtained from ATCC. Non-ALT cell lines were obtained from the ATCC. The HeLa cell line (subclone HeLa1.3) was described previously [56].

### Sequence analysis of *ATRX*, *DAXX*, and *H3.3* genes

Primers were synthesized by Invitrogen (San Diego, CA) to cover all exons of *ATRX*, *DAXX*, and *H3F3A1* (H3.3). PCR was performed in 5 µl reactions containing 1× PCR Buffer (67 mM Tris-HCl, pH 8.8, 6.7 mM MgCl2, 16.6 mM NH4SO4, 10 mM 2-mercaptoethanol), 1 mM dNTPs (Invitrogen, San Diego, CA), 1 µM forward and 1 µM reverse primers, 6% DMSO, 2 mM ATP,0.25 U Platinum *Taq* (Invitrogen, San Diego, CA) and 3 ng DNA. Reactions were carried out in 384-well ABI 9700 thermocyclers (Applied Biosystems, Foster City, CA) using a touchdown PCR protocol: 1 cycle of 96°C for 2 min; 3 cycles of 96°C for 10 sec, 64°C for 10 sec, 70°C for 30 sec; 3 cycles of 96°C for 10 sec, 61°C for 10 sec, 70°C for 30 sec; 3 cycles of 96°C for 10 sec, 58°C for 10 sec, 70°C for 30 sec; 41 cycles of 96°C for 10 sec, 57°C for 10 sec, 70°C for 30 sec; 1cycle of 70°C for 5 min. The PCR products were evaluated for presence and size by electrophoresis on 2% agarose gels and sequenced as described (refs. 25 and 57 [25], [57]). All failed PCRs were repeated at least two more times.

### Targeted sequencing of candidate ALT genes

Coding regions of 299 genes were amplified from genomic DNA of 15 samples. Following PCR amplification, products were normalized and pooled prior to emulsion PCR amplification of single stranded DNA and 454 pyrosequencing. Sequencing was performed using a FLX Titanium machine at Agencourt Bioscience Corp. (Beverly MA), to a depth of 20× coverage per sample per amplicon (attempted). Assembly and mapping of reads was performed using AVA software.

### Immunoblotting

Immunoblotting was performed using standard procedures on whole cell extracts. ATRX was detected primarily with A301-045 (Bethyl Labs) or with HPA001906 (Sigma Aldrich). DAXX was detected with A301-353A (Bethyl Labs). Immunoblotting for CHK2-P was done with CHK2-T69 (Cell Signaling) and total CHK2 was detected with clone 7 Ab (Millipore). Shelterin components were analyzed as described previously [56].

### Immunofluorescence

Cells grown on glass coverslips were fixed with 3% paraformaldehyde for 10 min at room temperature, washed three times with PBS, and permeabilized with 0.5% Triton X-100 in PBS for 10 min on ice. Cells were then washed four times with PBS and blocked for 30 min at room temperature with PBG (0.2% cold water fish gelatin (Sigma), 0.5% BSA, in PBS). Primary antibodies recognizing ATRX and DAXX (Bethyl Laboratories, Inc., see above) were diluted in PBG and incubated on cells for 1.5 h at 37°C. Following three washes with PBG, cells were incubated with FITC-conjugated donkey anti-rabbit secondary antibody (Life Technologies), diluted in PBG, for 30 min at 37°C. Cells were washed three times with PBS and counterstained with 4′,6′-diamidino-2-phenylindole (DAPI).

### Quantitative RT–PCR

RNA was extracted from cell lines using the RNeasy kit (Qiagen). 0.5 µg of RNA was reverse transcribed using RT-Advantage kit (Clontech) in a volume of 20 µl according to the manufactures instructions. Following reverse transcription 180 µl of water was added and 5 µl was used for qPCR with specific primers and SYBR green mix (Applied Biosystems). Primers: DAXX 5′: AGACGGTTTCTGAGCATCATC; DAXX 3′: AGAGGAGCTAGGGGCTTCTG; TERT 5′: GCCTTCAAGAGCCACGTC; TERT 3′: CCACGAACTGTCGCATGT.

### Analysis of copy number alterations

Affymetrix 6.0 SNP data were generated at the Broad Institute for 22 ALT cell lines and 6 single cell ALT (JFCF-6/T.1R) and non-ALT (HCT116) cell line clones. Single cell clones were generated by fluorescence activated cell sorting (FACS) sorting into 96 well plates. Normalized copy number estimates (log2 ratios) were made and segmented by the Circular Binary Segmentation algorithm as previously described [58]. The GISTIC 2.0 algorithm was performed as previously described on the resulting segmented copy number data from the 22 ALT lines [39], [59]. The boundaries of the peak amplified and deleted regions identified by GISTIC 2.0 were set with at least 95% confidence to include the target gene(s).

### Functional analysis of DAXX and ATRX

SV40-transformed BJ fibroblasts at PD 38 were infected with either *ATRX*- or *DAXX*-specific shRNAs (Sigma) with the following target sequences: ATRXsh589: GCAGATTGATATGAGAGGAAT; ATRXsh590: CGACAGAAACTAACCCTGTAA; ATRXsh592: CCGGTGGTGAACATAAGAAAT; DAXXsh800 : GAAGGGATGGACTAAGCTAAT; DAXXsh801: TCACCATCGTTACTGTCAGAA; DAXXsh802: GCCACACAATGCGATCCAGAA. LacZ-specific shRNA was used as negative control, while infection with hTERT was used as a positive control. Cells were selected with puromycin (0.5 µg/ml) for 2 days, and at least 10^6^ cells of each sub-line were maintained in culture for 110 days. Cultures were split at <80% confluence, and growth medium was replaced every 3 days. Cells were counted at each passage and cumulative PDs were recorded. The suppression of *ATRX* in HeLa1.3 cells was achieved with ATRXsh592.

### T-SCE assay for Telomeric Recombination

Cells were grown in 10 µM BrdU∶BrdC (3∶1) for 16 hr with the addition of 0.1 µg/ml colcemid (Roche) for the final 2 h. Slides were treated with 0.5 mg/ml RNAse A for 10 min at 37°C, stained with 0.5 µg/ml Hoechst 33258 in 2× SSC for 15 min at room temperature, and exposed to 365-nm UV light (Stratalinker 1800 UV irradiator, 5400 J/m^2^). Following digestion with Exonuclease III (Promega, 10 U/µl, 2×10 min) at room temperature, slides were dehydrated through an ethanol series (70%, 90%, 100%) and incubated sequentially with TAMRA-TelG 5′-[TTAGGG]_3_-3′ and FITC-TelC 5′-[CCCTAA]_3_-3′ probes at room temperature. The percentage of chromosome ends displaying telomeric sister chromatid exchanges (T-SCEs) was calculated from four independent experiments.

### SKY

Cells were treated with colcemid for 1 hr, harvested, pelleted, re-suspended in a hypotonic solution of 0.075 M KCl for 18 min, fixed in Carnoy's fixative (3∶1 methanol∶glacial acetic acid), and washed four times with Carnoy's fixative. All fixed samples were spread on slides for staining or hybridization. Chromosomes were stained with Giemsa or Hoechst to visualize chromosomal abnormalities. Spectral karyotyping (SKY) was performed on mitotic samples according to the SkyPaint DNA kit H-5 for human chromosomes procedure (Applied Spectral Imaging, SKY000029) and imaged on a Nikon Eclipse E6000 microscope equipped with the SD300 Spectracube and Spectral Imaging acquisition software.

### G2/M checkpoint analysis

To assess G2/M checkpoint initiation, subconfluent cells were exposed to γ-irradiation and harvested at 1 hr with non-irradiated cells for mitotic index measurement by flow cytometry. Briefly, cell pellets were fixed in 70% ethanol, washed twice with cold PBS, and permeabilized with 0.25% Triton X-100 in PBS for 15 min on ice. Cells were then rinsed with PBS containing 1% BSA and stained with anti-phospho-histone H3 S10 antibody (Cell Signaling) for 1.5 hr at room temperature. Following two washes with PBS containing 1% BSA, cells were stained with a FITC-conjugated donkey anti-mouse secondary antibody (Life Technologies) for 30 min at room temperature. Cells were washed twice with PBS and then stained with propidium iodide (25 µg/ml) in the presence of 0.1 mg/ml RNase. Flow cytometry was performed on a BD Biosciences FACSCalibur, and the percentage of mitotic cells was determined as those that were FITC-positive and contained 4N DNA content. To assess maintenance of the G2/M checkpoint, nocodazole (1 µg/ml) was added 1 hr after irradiation, and cells were incubated at 37°C for 16 hr. Cells were then harvested and processed as described above. To account for differences in the rate of cell cycle progression between cell lines, the mitotic index of irradiated cells was normalized to that of non-irradiated cells treated with nocodazole for 16 hr.

### Micronucleation

Asynchronously growing cells were fixed and examined for the presence of micronuclei after DAPI staining for DNA. Data were obtained from three independent experiments with >100 nuclei examined in each.

### C-circle assay

For the C-circle assay, DNA from cell lines was isolated using the DNeasy Blood and Tissue Qiagen kit, digested with *Hinf*I and *Rsa*I, and quantified using Hoechst fluorimetric analysis. DNAs were diluted to approximately 3 ng/µl, quantified again, and the dilutions were adjusted to exactly 3 ng/µl. The assay was performed using 30 ng of quantified DNA as described by Henson et al. [8] using dot-blotting with an end-labeled [AACCCT]4 telomeric oligonucleotide probe. C-circle values in Table 1 were derived from three independent DNA preparations for each cell line.

### DSB repair assays

Cells grown on glass coverslips were irradiated with 0.5 Gy IR and incubated at 37°C for 1 hr or 24 hr. Irradiated and non-irradiated cells were fixed with 3% paraformaldehyde for 10 min at room temperature, washed three times with PBS, and permeabilized with 0.5% Triton X-100 for 10 min on ice. Cells were then washed four times with PBS and blocked for 30 min at room temperature with PBG (0.2% cold water fish gelatin (Sigma), 0.5% BSA, in PBS). Primary 53BP1 (Novus) antibody was diluted in PBG and incubated on cells for 1 hr at 37°C. Following three washes with PBG, cells were incubated with FITC-conjugated donkey anti-rabbit secondary antibodies (Life Technologies), diluted in PBG for 30 min at 37°C. Cells were washed three times with PBS and counterstained with 4′,6′-diamidino-2-phenylindole (DAPI). The percentage of cells containing >10 53BP1 foci per nucleus was calculated from three independent experiments.

For the PFGE assay, subconfluent cells were irradiated with 100 Gy IR and incubated at 37°C for 24 hr. Irradiated and non-irradiated cells were washed once with PBS, trypsinized, and counted. One million cells per sample (in duplicate) were centrifuged and washed a second time with PBS. Cell pellets were resuspended in 50 µl 10 mM Tris pH 7.2, 20 mM NaCl, 50 mM EDTA and pre-warmed at 55°C for 10 min. The cell suspension was then mixed with 50 µl of 2% agarose/PBS at 55°C and solidified in plastic molds (BioRad). Agarose plugs were incubated with 1 mg/ml Proteinase K, 0.2% sodium deoxycholate, 1% N-lauroylsarcosine-sodium salt, 100 mM EDTA, pH 8.0 for 20 hr at 50°C and then washed four times for 1 hr with 20 mM Tris/HCl, 50 mM EDTA, pH 8.0. Duplicate plugs were irradiated with 600 Gy IR, and all samples were equilibrated in 0.5× TBE for 30 min. Agarose plugs were inserted into the wells of a 1% agarose gel and sealed with 1% agarose. PFGE was carried out using a CHEF-II apparatus in 0.5× TBE buffer at 6 V/cm with pulse times of 70 sec (15 hr) and 120 sec (11 hr). Following electrophoresis, the gel was stained with 0.5 µg/ml ethidium bromide in TBE for 30 min.

## Supporting Information

Figure S1 C-circle assay on the ALT cell line panel. Example of dot-blot results of C-circle assay on ALT lines and non-ALT negative control (BJ/hTERT/SV40, blue). Amounts of DNA used for the assay are indicated.
(TIF)

Figure S2 Lack of ALT-mediated immortalization upon suppression of ATRX or DAXX. A, Q-RT PCR assay for the expression of hTERT mRNA in the indicated cell lines. B, C-circle assay on the indicated cell populations. JFCF-6/T.1F is a non-ALT cell line that serves as a negative control. The positive control is JFCF-6/T.1J/1-3C. C, Q-RT PCR assay for expression of DAXX mRNA in the indicated cell populations. D, Proliferation of SV40-transformed BJ fibroblasts infected with the indicated hTERT or shRNA retroviruses.
(TIF)

Figure S3 Immunoblotting for shelterin components in ALT. Immunoblots for the indicated shelterin components in whole cell extracts of the indicated cell lines.
(TIF)

Figure S4 Analysis of TERRA levels in ALT. Northern blots of total RNA from the indicated cell lines probed for TERRA with a C-strand telomeric repeat probe. The ethidium bromide staining pattern of the gel is shown with the ribosomal RNAs indicated. The bar graphs show the relative expression levels of TERRA derived from 3–5 independent experiments and standard deviations. Telomerase-positive controls, BJ/hTERT/SV40 and HeLa, are shown in blue.
(TIF)

Figure S5 Regions of recurrent amplification and deletion in 22 ALT lines. GISTIC 2.0 analysis of deletion (blue lines, left panel) and amplification (red lines, right panel) events identifies significantly recurrent peak regions (top 25 labeled by cytoband). False discovery rates (q-values; x-axis) are plotted by genomic position (y-axis) with the green line indicating the 0.25 cut-off for significance.
(TIF)

Figure S6 DDR signaling in ALT lines. Immunoblot showing phosphorylation of CHK2 before and after treatment with IR in the indicated ALT lines and the RPE/hTERT control (blue).
(TIF)

Figure S7 Example of assay for DSB repair kinetics. The graph shows the % of cells with >10 53BP1 foci scored by IF on >100 cells. The indicated cell lines were either not treated with IR, treated with 0.5 Gy and incubated for 1 hr or for 24 hr. Data from two experiments were averaged. Control telomerase-positive cell lines (blue) show disappearance of IR-induced foci. Each of the ALT lines has residual IR-induced DSBs at 24 hr.
(TIF)

Table S1 *ATRX* exons deleted in the analyzed ALT lines.
(XLSX)

Table S2 List of sequenced genes.
(XLS)

Table S3 Sequence alterations in the set of 299 genes analyzed in the ALT cell lines.
(XLSX)
