# Supplementary figures and images for: Loss of ATRX, Genome Instability, and an Altered DNA Damage Response Are Hallmarks of the Alternative Lengthening of Telomeres Pathway
Source: PLoS Genet. 2012 Jul 19;8(7):e1002772. doi: 10.1371/journal.pgen.1002772 (PMC3400581; doi:10.1371/journal.pgen.1002772)

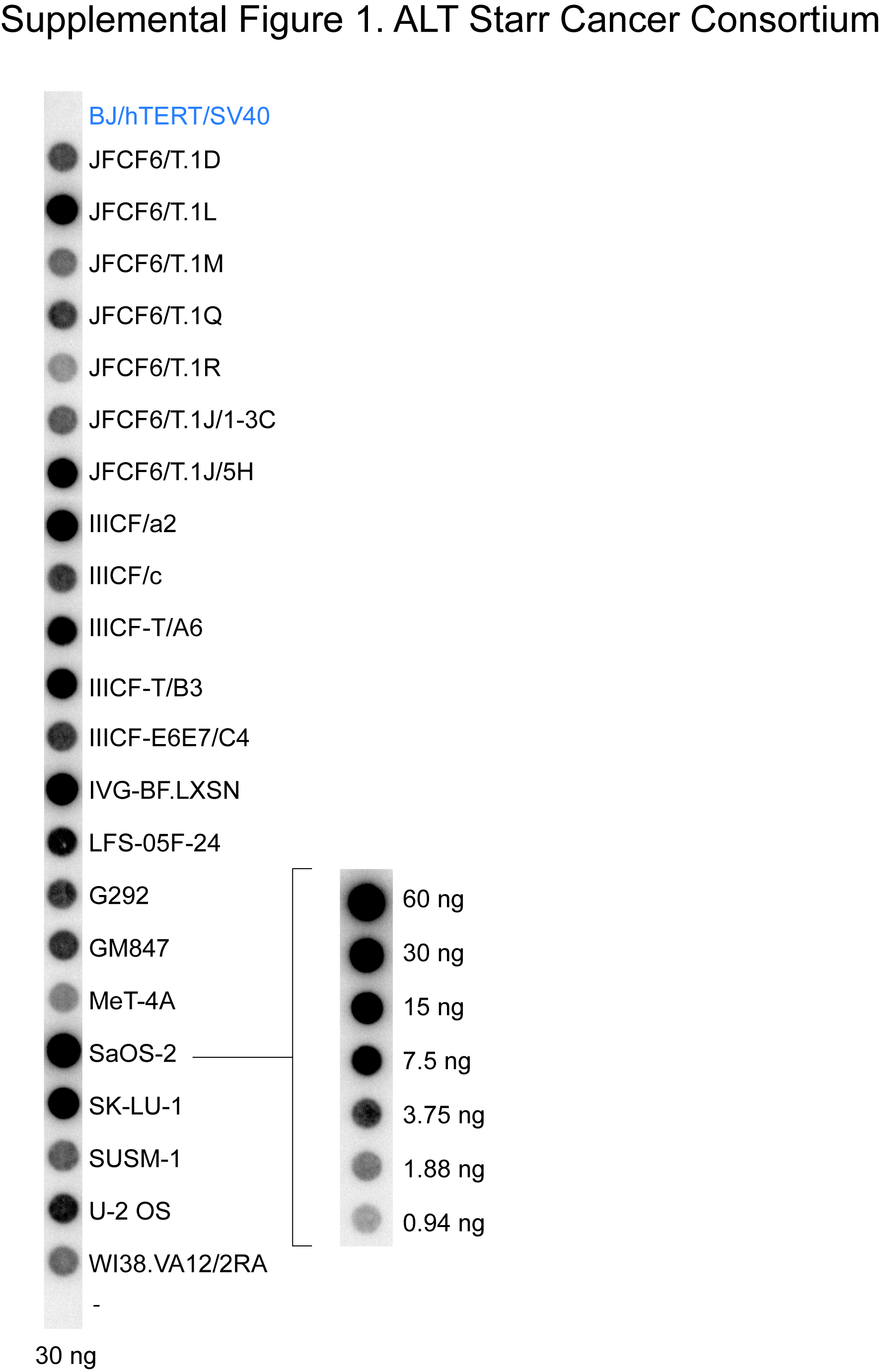

Supplement: Figure S1 — C-circle assay on the ALT cell line panel. Example of dot-blot results of C-circle assay on ALT lines and non-ALT negative control (BJ/hTERT/SV40, blue). Amounts of DNA used for the assay are indicated. (TIF) [file pgen.1002772.s001.tif]

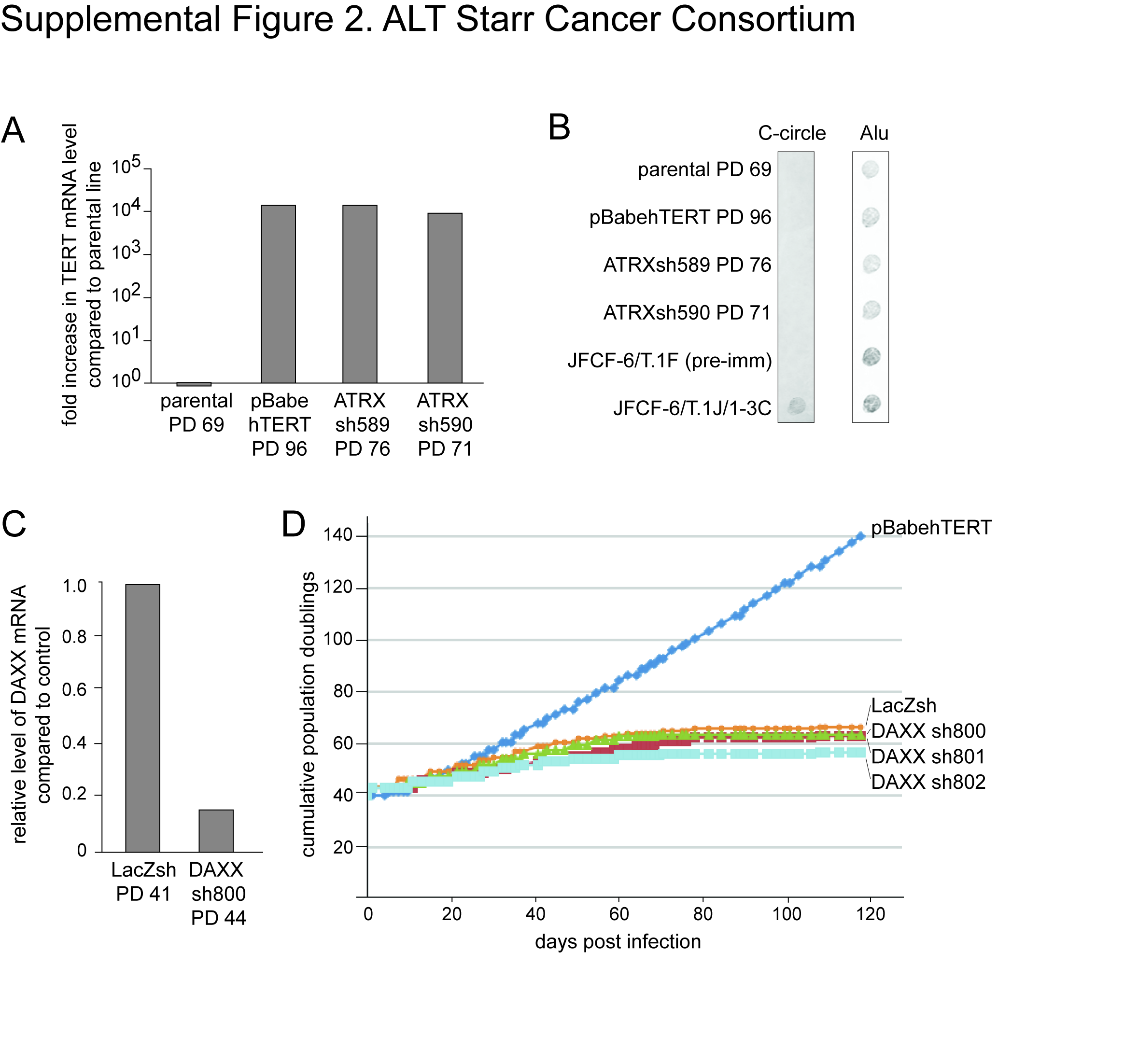

Supplement: Figure S2 — Lack of ALT-mediated immortalization upon suppression of ATRX or DAXX. A, Q-RT PCR assay for the expression of hTERT mRNA in the indicated cell lines. B, C-circle assay on the indicated cell populations. JFCF-6/T.1F is a non-ALT cell line that serves as a negative control. The positive control is JFCF-6/T.1J/1-3C. C, Q-RT PCR assay for expression of DAXX mRNA in the indicated cell populations. D, Proliferation of SV40-transformed BJ fibroblasts infected with the indicated hTERT or shRNA retroviruses. (TIF) [file pgen.1002772.s002.tif]

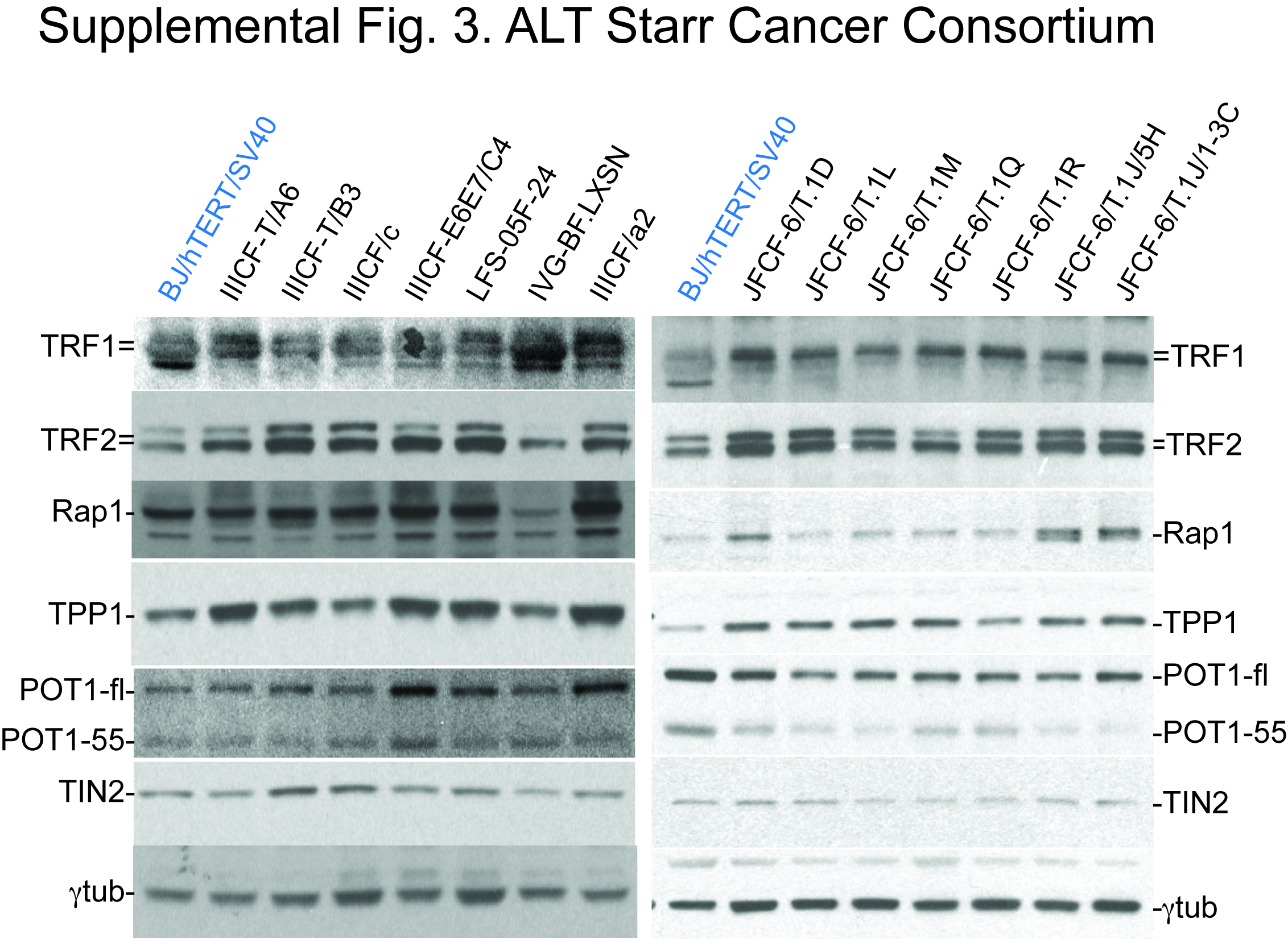

Supplement: Figure S3 — Immunoblotting for shelterin components in ALT. Immunoblots for the indicated shelterin components in whole cell extracts of the indicated cell lines. (TIF) [file pgen.1002772.s003.tif]

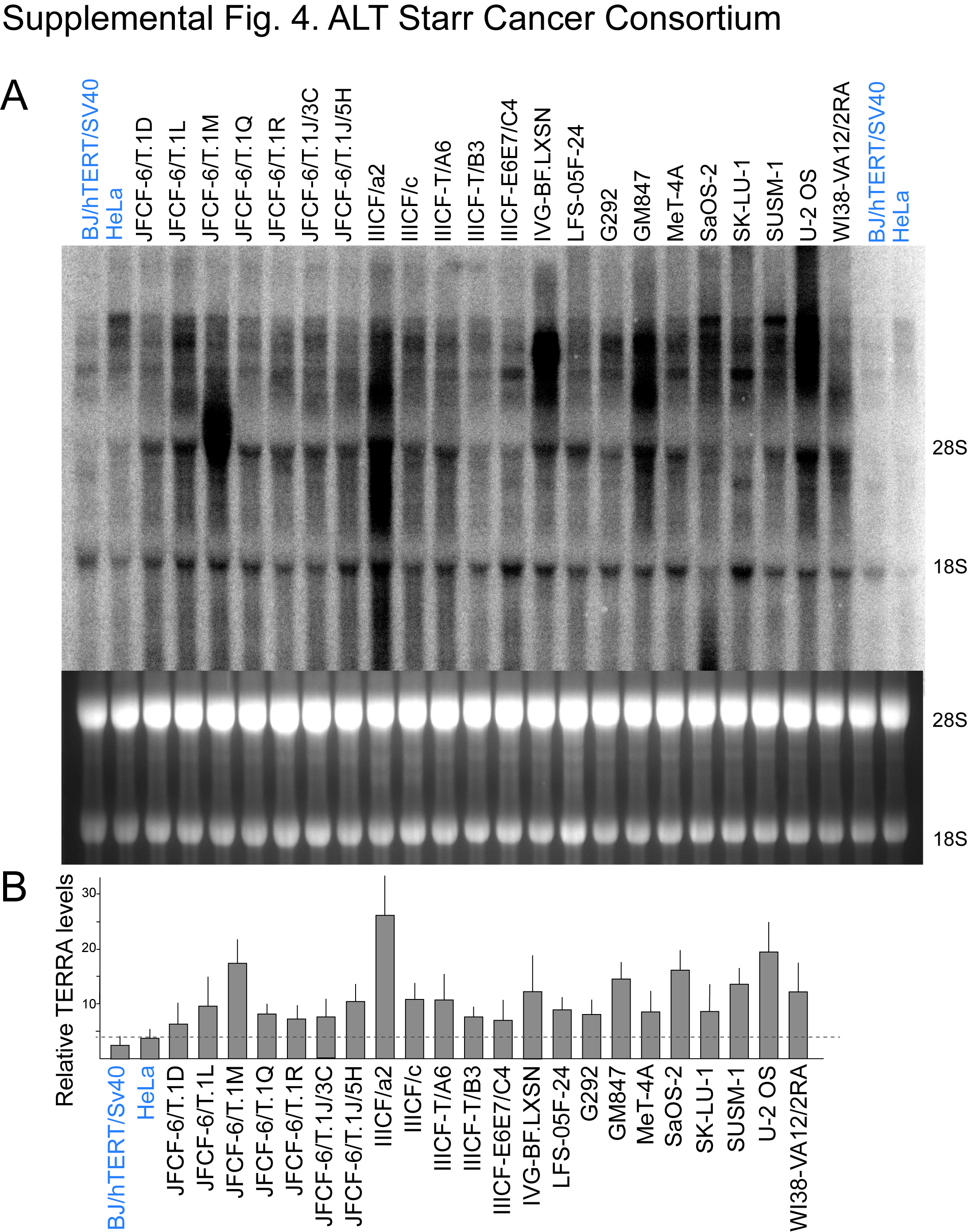

Supplement: Figure S4 — Analysis of TERRA levels in ALT. Northern blots of total RNA from the indicated cell lines probed for TERRA with a C-strand telomeric repeat probe. The ethidium bromide staining pattern of the gel is shown with the ribosomal RNAs indicated. The bar graphs show the relative expression levels of TERRA derived from 3–5 independent experiments and standard deviations. Telomerase-positive controls, BJ/hTERT/SV40 and HeLa, are shown in blue. (TIF) [file pgen.1002772.s004.tif]

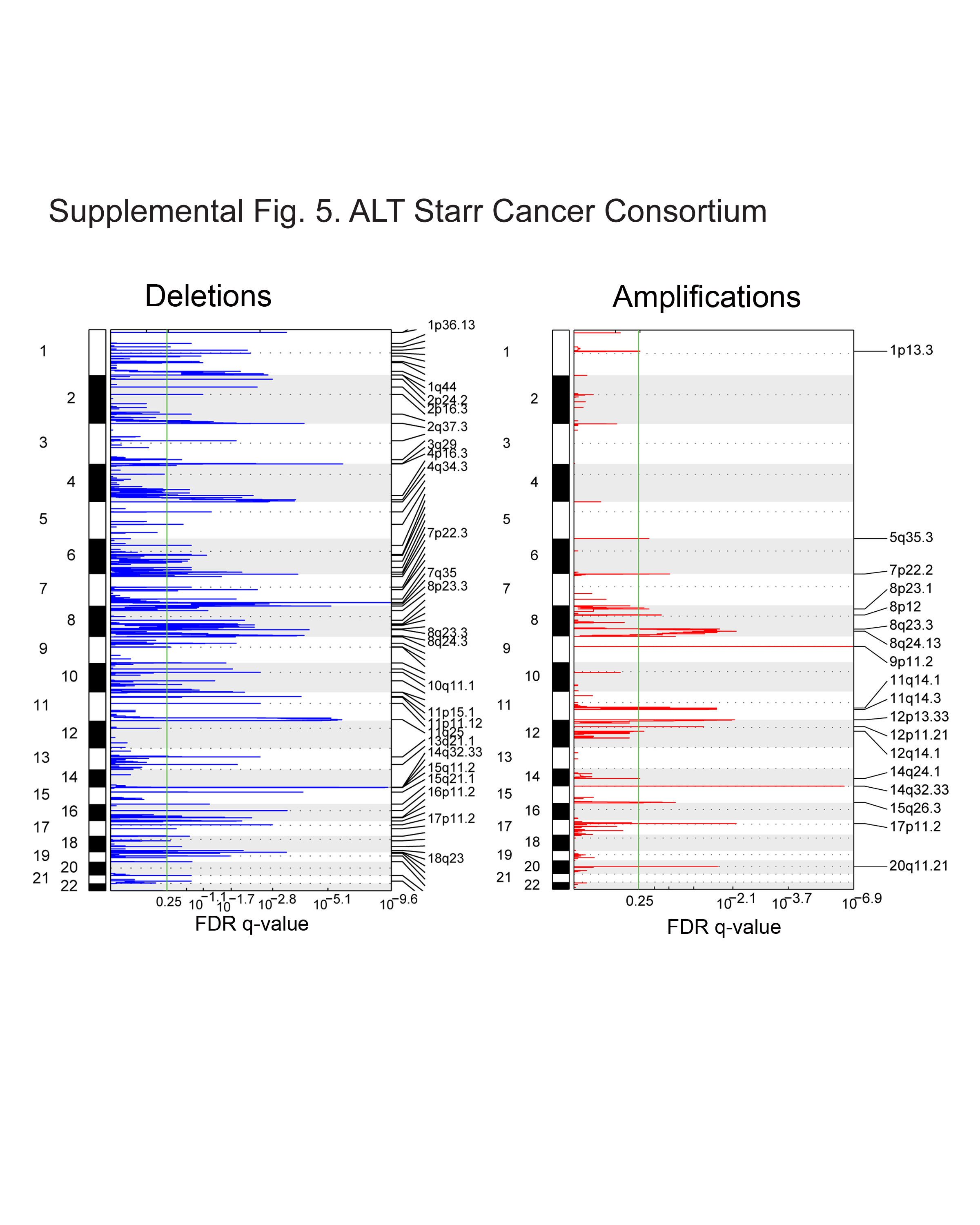

Supplement: Figure S5 — Regions of recurrent amplification and deletion in 22 ALT lines. GISTIC 2.0 analysis of deletion (blue lines, left panel) and amplification (red lines, right panel) events identifies significantly recurrent peak regions (top 25 labeled by cytoband). False discovery rates (q-values; x-axis) are plotted by genomic position (y-axis) with the green line indicating the 0.25 cut-off for significance. (TIF) [file pgen.1002772.s005.tif]

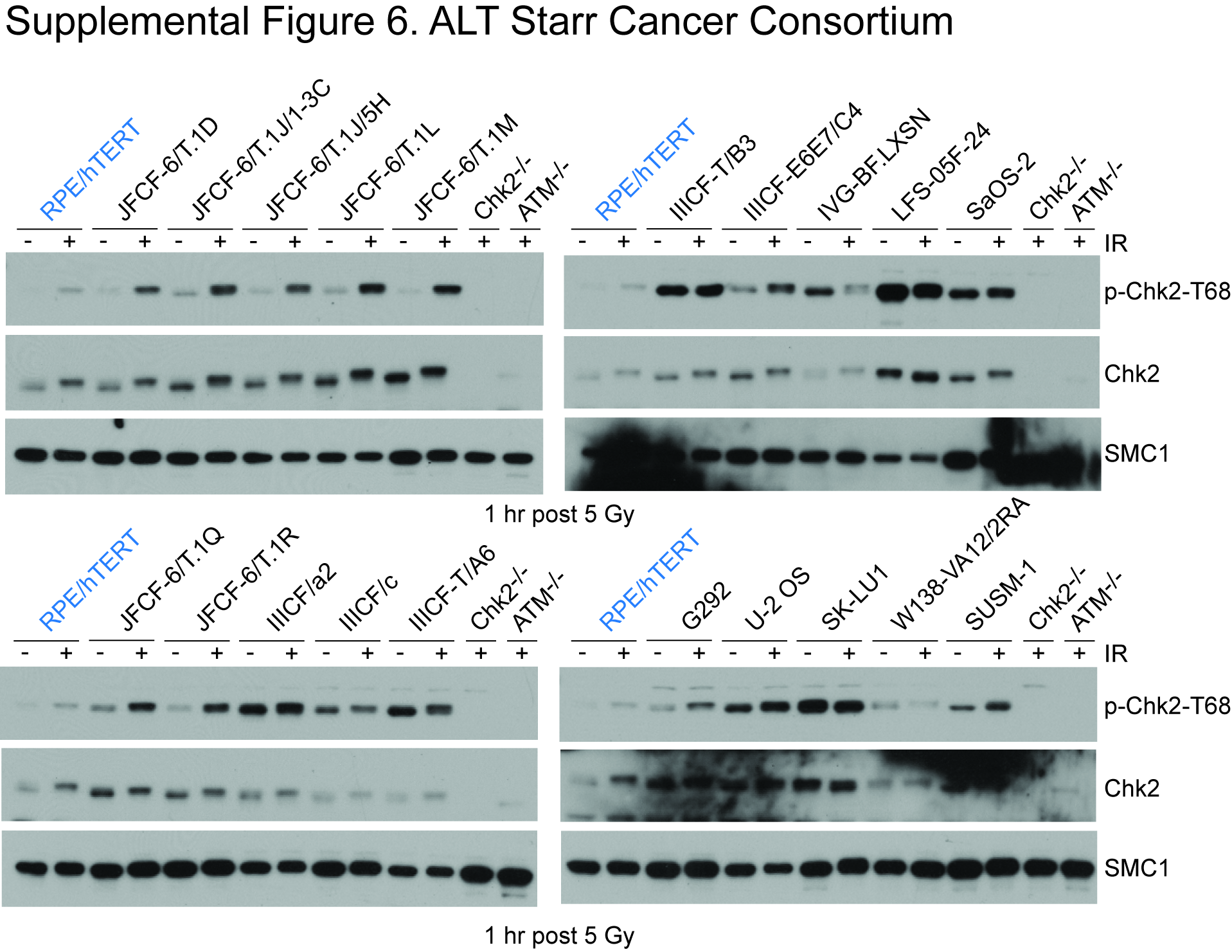

Supplement: Figure S6 — DDR signaling in ALT lines. Immunoblot showing phosphorylation of CHK2 before and after treatment with IR in the indicated ALT lines and the RPE/hTERT control (blue). (TIF) [file pgen.1002772.s006.tif]

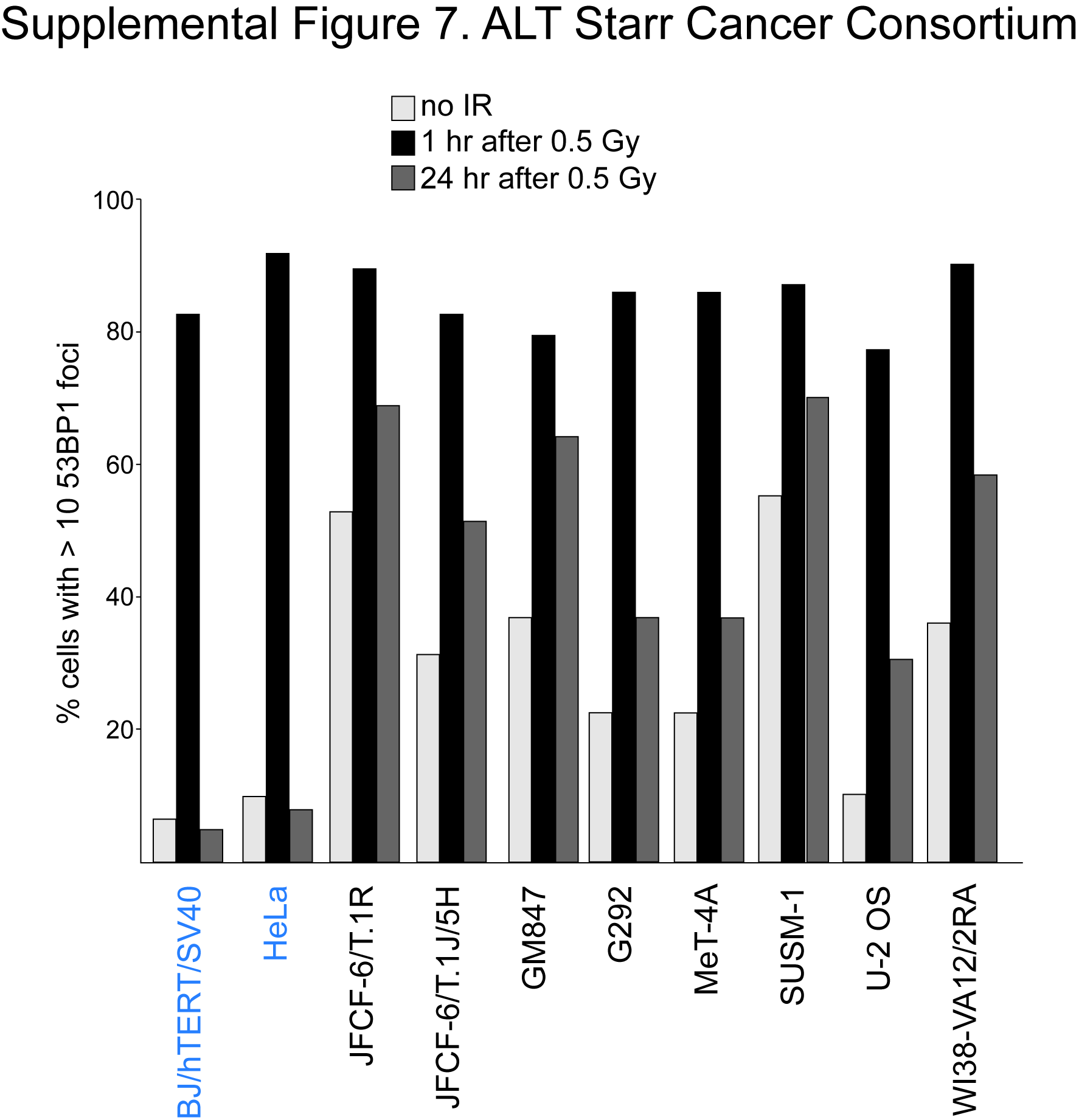

Supplement: Figure S7 — Example of assay for DSB repair kinetics. The graph shows the % of cells with >10 53BP1 foci scored by IF on >100 cells. The indicated cell lines were either not treated with IR, treated with 0.5 Gy and incubated for 1 hr or for 24 hr. Data from two experiments were averaged. Control telomerase-positive cell lines (blue) show disappearance of IR-induced foci. Each of the ALT lines has residual IR-induced DSBs at 24 hr. (TIF) [file pgen.1002772.s007.tif]
